# Survival analysis of Sudanese oral squamous cell carcinoma patients with field of cancerization

**DOI:** 10.1186/s12885-024-12197-7

**Published:** 2024-04-15

**Authors:** Yousif Eltohami, Ahmed Suleiman

**Affiliations:** Oral and Maxillofacial Surgery, Faculty of Dentistry, University of Khartoum, Khartoum, Sudan

**Keywords:** Oral squamous cell carcinoma, Wide field of cancerization, Recurrence, Second primary tumour, Survival rates

## Abstract

**Background:**

The late presentation and diagnosis of OSCC account for the large number of patients with the advanced form of the disease. In Sudan, cases with delayed presentation, particularly those with risk factors such as Toombak dipping and alcohol consumption, frequently present with extensive lesions and a wide area of Field cancerization which characterized by the presence of genetic and epigenetic changes in histologically normal-appearing tissues, and have increased risk for recurrent and second primary tumors. This necessitates more aggressive treatment and is usually associated with poorer outcomes. The present study aims to investigate the survival of oral squamous cell carcinoma patients with a wide field of cancerization.

**Methods:**

This prospective longitudinal study includes ninety-three oral cancer patients with extensive fields of cancerization who underwent surgical treatment at Khartoum Teaching Dental Hospital (KTDH) conducted from 2019 to 2023. These patients were regularly assessed for clinical changes such as recurrence, the development of second primary tumours, and overall survival over a period of one year.

**Results:**

Out of the 93 patients, 57 (61.3%) were males, and 36 (38.7%) were females. The majority of the patients (82%) had stage IV tumours, and 62.3% had nodal metastasis. Twenty-eight (30%) patients developed recurrences, and 14 (15%) developed second primary tumours. The overall one-year survival rate was 89%, and all deceased patients passed away within 12 months. The survival rate for patients with different types of recurrences varied, with patients who had regional, local, and locoregional recurrences having survival rates of 87%, 74%, and 72%, respectively. Patients who did not experience a recurrence had a one-year survival rate of 92%. Patients who developed second primary tumours had an 86% survival rate. The survival rates for OSCC patients at stages III, IVa, and IVb were 90%, 90%, and 71%, respectively.

**Conclusion:**

In this study, 62% of patients had nodal metastasis, 30% developed recurrence, and 15% developed second primary tumours. The overall one-year survival rate was 89%, although the development of recurrences and second primary tumours had a negative impact on the survival rate.

## Introduction

Globally, head and neck cancer (HNC) remains a leading cause of death [1]. Among these cancers, squamous cell carcinoma (SCC) is the most common malignancy in the oral epithelium, ranking as the sixth most common cancer in the world [2]. In 2017, Salah et al. reported that oral cancer in Sudan was the seventh most common cancer among males and the eighth most common among females [3]. The term “field of cancerization” is used to describe neoplastic processes at different regions that can develop independently. The presence of multiple primary and secondary tumours may result from the susceptibility of epithelial cells in various sites to carcinogens or the lateral spread of premalignant epithelial cells, ultimately leading to the development of new tumours [4]. Brennan et al., using the P53 assay, found clonally related neoplastic cells in the surgical margins of over 50% of OSCC lesions, even when these margins appeared negative under the microscope [5].

Variations in survival rates are recognized due to differences in initial clinical characteristics, the effectiveness of registration, the patient’s socio-economic status, and the presence of an efficient healthcare system [6]. Several factors influence oral cancer survival, including tumour clinical staging at presentation, tumour size and thickness, histopathological grading, lymphovascular invasion (LVI) and perineural invasion (PNI), the status of resection margins, regional and distant metastasis, locoregional recurrence, and the treatment modalities received [7]. The primary survival rate for OSCC is typically around 45%, and it drops to less than half when there is nodal and distant metastasis [8]. Studies have shown that OSCC survival is significantly shorter in late-stage patients, with survival rates ranging from 80% for stage I to only 10% for stage IV [9]. This study aims to investigate the one-year overall survival rates in OSCC patients with extensive fields of cancerization.

## Materials and methods

This prospective longitudinal hospital based study (2019–2023) included patients who underwent surgical treatment at Khartoum Teaching Dental Hospital (KTDH) in Sudan. The patients were selected after a thorough clinical assessment of their oral cavity and head & neck regions for clinically suspicious ulcerative lesions associated with extensive mucosal changes. The study excluded patients with recurrent or second primary lesions and those who received neoadjuvant radiation or chemotherapy. Recurrence variables were assessed for the presence of recurrence, the type of recurrence, the date of recurrence, and its clinical presentation. A second primary tumour (SPT) was reported when a new tumour developed more than 2 cm away from the primary lesion. Local recurrence after surgery was described if the disease recurred within 2 cm of the original lesion [10].

The follow-up period lasted one year and included inspections, photography (digital camera), palpation of cervical lymph nodes, and CT scans of the head and neck region. Biopsies were repeated if a new, recurrent whitish or reddish patch or an ulcerative lesion appeared or if there was a suspicious area with a change in the normal colour and texture of the mucosa. Fine-needle aspiration cytology and computed tomography with contrast were used for the diagnosis of any suspicious regional nodal recurrence. The follow-up period ended if the patient was not punctual with follow-up, was lost to follow-up, developed distant metastasis, or died.

### Statistical analysis

Data were entered into a computer database using SPSS version 26. Statistical analysis was performed at a 95% confidence level with a significance level of 0.05. Kaplan-Meier analysis was used to determine survival rates. Multivariate analysis and logistic regression were performed for better predictive accuracy and to control for confounding factors, with results presented as odds ratios and p-values.

## Results

The study included 93 patients (186 specimens) with OSCC who were diagnosed and surgically treated at KTDH. The patients were 57 (61.3%) males and 36 (38.7%) females. The age of the patients ranged between 25 and of 80 years, and the mean age of the patients on diagnosis was 57.52 ± 12.506 years. Regarding the social habits, 38 (39.9%) of the patients were snuff dippers, 19 (20.4%) were tobacco smokers and 14 (15.1%) were alcohol drinkers (Table 1).

### Clinical characteristics of the lesions

**Fig. 1:**
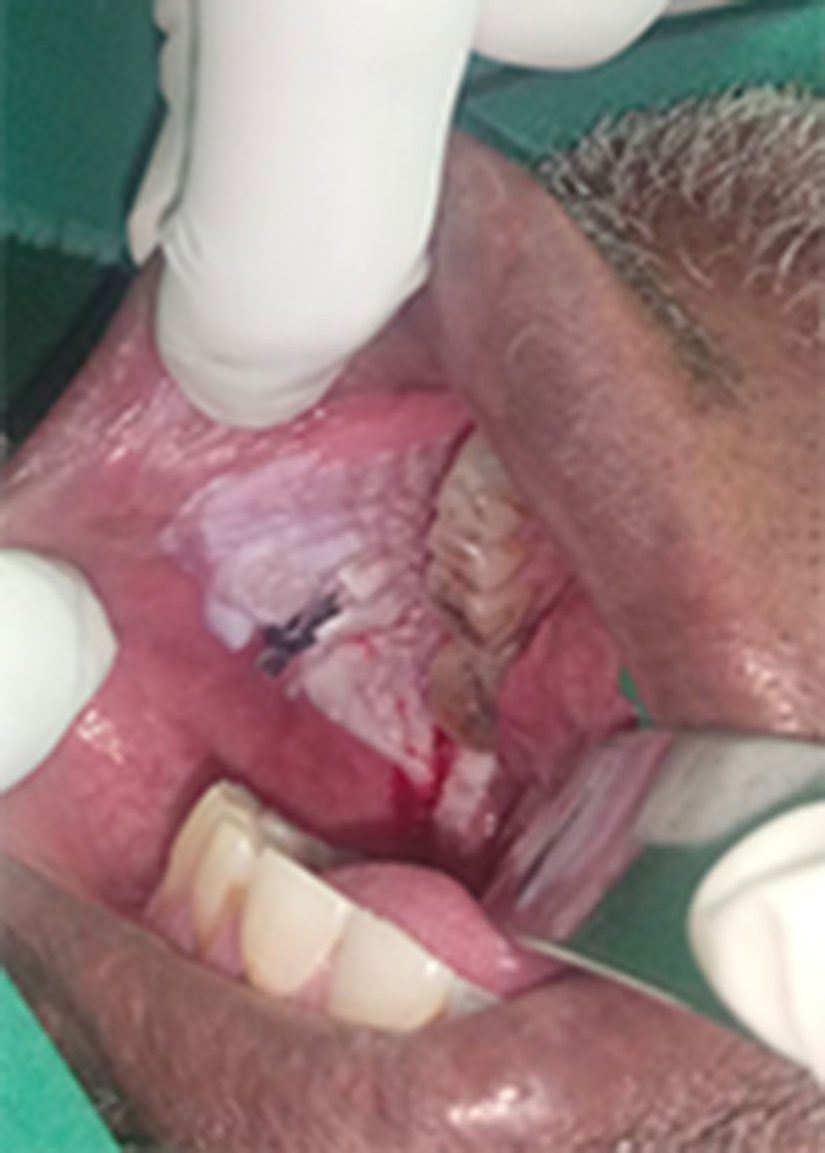
Another OSCC patient with mucosal changes (grizzle mixed dark and white discoloration) of wide field of cancerization

**Fig. 2:**
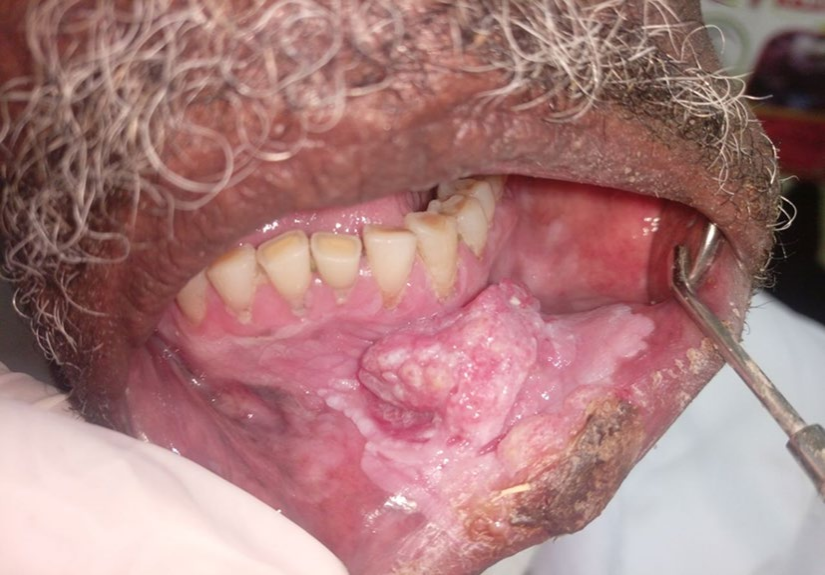
An OSCC patient with mucosal changes of wide field of cancerization (cotton white discoloration)

Ninety-two patients had multiple sites involved in the oral cavity with overlapping of the lesions and the regions involved. The gingivobuccal and the gingivolabial mucosae were the most reported sites involved accounting for 74.2% (69) and 62.3%(58) of the cases respectively (Table 2). Regarding the clinical presentation of OSCC lesions, all the 93 (100%) cases presented with an ulcer as a complaint, and 83 (89.2%) of the lesions were surrounded by different mucosal changes. Overlapping presentations were described in 44 (47.3%) of the lesions that were surrounded by erythematous patches, accentuating the clinical presentation of wide field of changes (Table 3). Eighty-three (89.2%) of the lesions were surrounded by mucosal changes, of them 32(38%) surrounded by grizzle (mixed dark and white) (Fig. 1) and 21(26%) were surrounded by white smoke discoloration followed by 17(20%) and 13(16%) cotton white (Fig. 2) and Café au lait discolorations respectively. Fifty-four (58%) of the cases presented with ulcerative lesions ranging from around 2 to 4 cm. Of the series, 69 (74.2%) patients presented with advanced stage IVa (T4a + N0/N1/N2 + M0) tumors, 7 (7.5%) patients presented with stage IVb. Additionally,17 (18.2%) patients presented with stage III tumors, and there were no cases of early cancer i.e. stage I and stage II among the series (Table 4). Eighty-four per cent, 84% of the snuff dippers had multiple sites involvement. Of the 94% had a wide field change of mixed white and dark discoloration, and 71% showed erythematous patches within the field.

### Surgical treatment and adjuvant therapy

Of the series; 19 patients (20%) underwent maxillectomy, 53 (57%) patients underwent mandibular resection, 15 (16.1%) patients underwent hemi-glossectomy, and seven (7.5%) patients underwent partial glossectomy. Ninety-one (97.8%) patients underwent modified comprehensive neck dissection; of them 23(24.7%) underwent also supraomohyoid ND on the contralateral side (Table 5). Thirteen (14.0%) of the patients received reconstruction, nine (9.7%) were reconstructed by nasolabial flap and four (4.3%) of the cases were reconstructed by deltopectoral flap. Seventy-eight (83.8%) patients received adjuvant therapy; concurrent chemoradiation therapy (CCRT) was given to 58 (62.4%) patients, and radiotherapy alone was given to 20 (21.5%) patients (Table 6).

### Postoperative histopathological characteristics

In 64 (68.8%) patients, the malignant tumor was well differentiated squamous cell carcinoma and in 15 (16.1%) patients the cancer was moderately differentiated carcinoma (Table 7). In 92 (98.9%) patients the tumor depth of invasion (TDI) was > 4 mm. The presence of histopathological dysplasia at the surgical margins in OSCC cases with wide field of cancerization was identified in only 13 (14.0%) of the cases, and all of them showed mild dysplasia. Extranodal extension (ENE) was identified in 43 (46.2%) of the cases. Fifty-eight (62.4%) of the cases showed positive pathological nodal involvement, and one third (37.6%) of the cases showed absence of histological nodal metastasis. Lymphovascular invasion was reported in 76 (81.7%) of the cases and similarly, perineural invasion was identified in 72 (77.4%) of the cases. The status of the surgical margins in 92 (98.9%) patients showed negative margins (Table 8).

### Recurrence and SPT characteristics

Twenty-eight (30%) of the patients developed recurrence, 17 (18.3%) of them had regional recurrence. Of the latter, 12 (71%) had recurrence on the non- operated contralateral side of the neck, and five (29%) developed recurrence in the operated neck side. Of the 28 cases with the recurrence, eight (8.6%) cases developed local recurrence, and three (3.2%) patients developed locoregional recurrence. With regard to the wide field of cancerous changes, 14 (15.1%) of the cases developed second primary lesions (Table 9). Multivariate cox regression analysis for recurrence and second primary tumors variables was showed in Tables 10 and 11.

### Survival rates

**Fig. 3:**
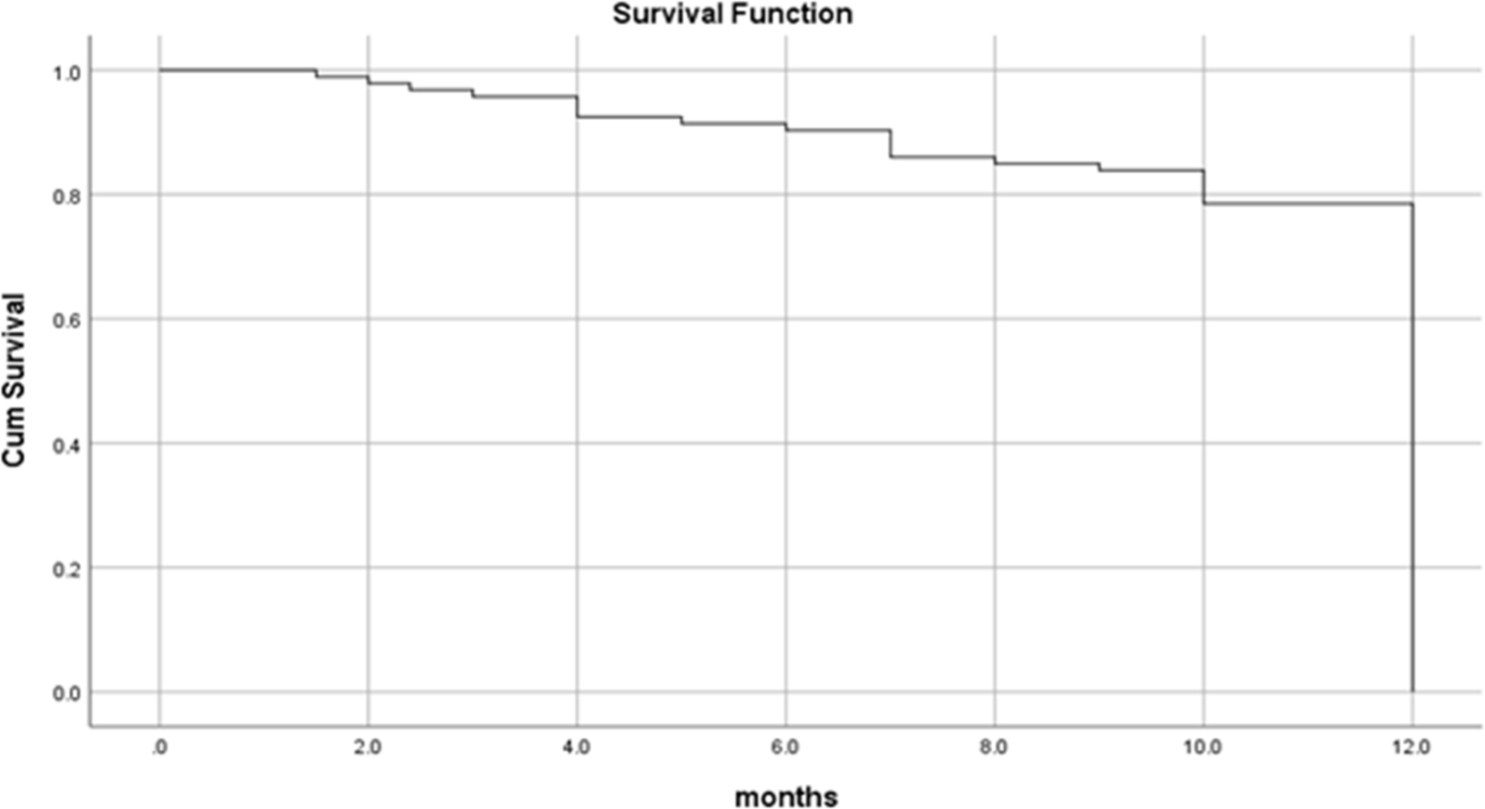
Kaplan Meier overall survival rate of the OSCC patients

**Fig. 4:**
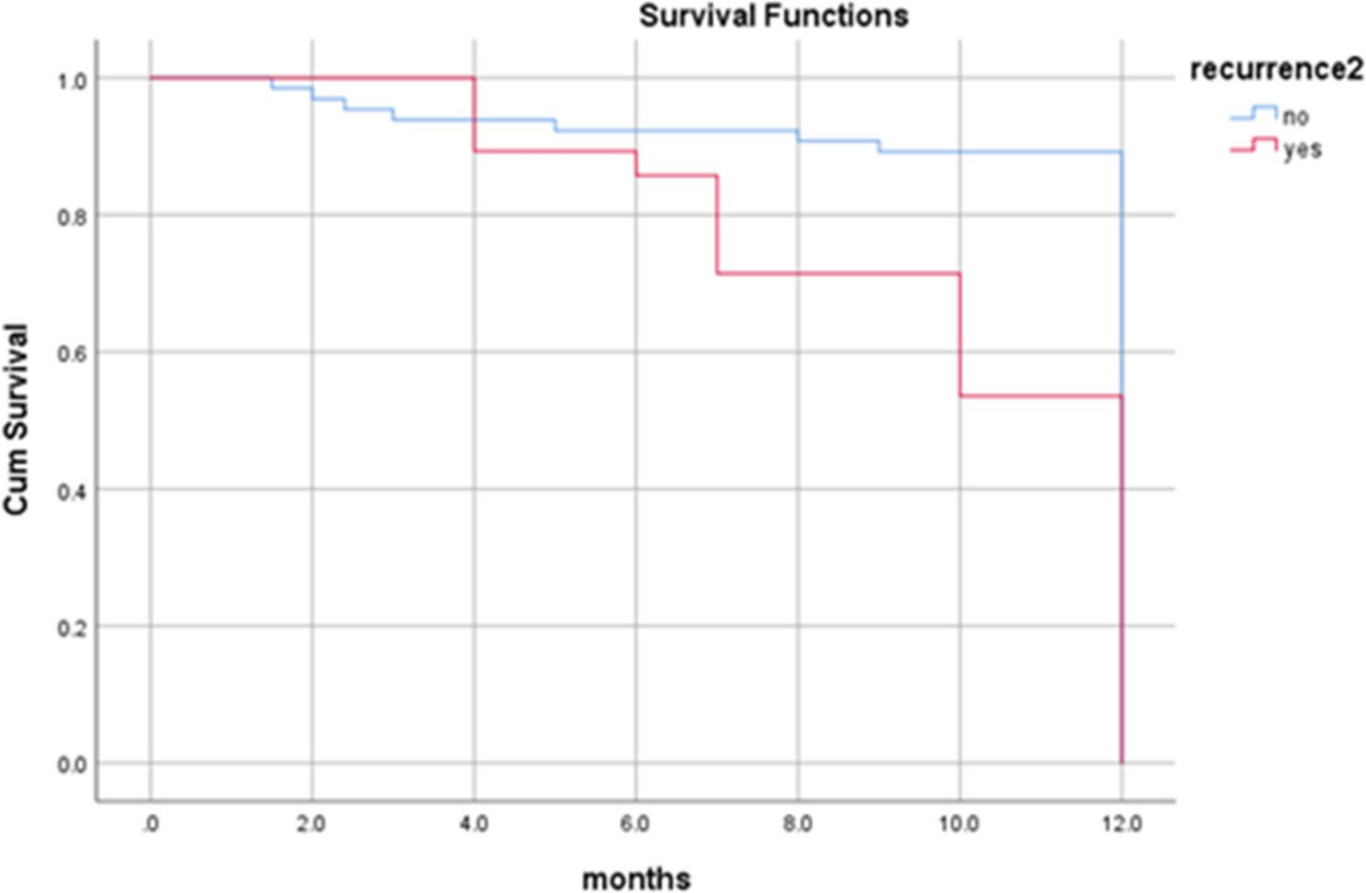
Kaplan Meier survival rate according to presence of the overall recurrences

**Fig. 5:**
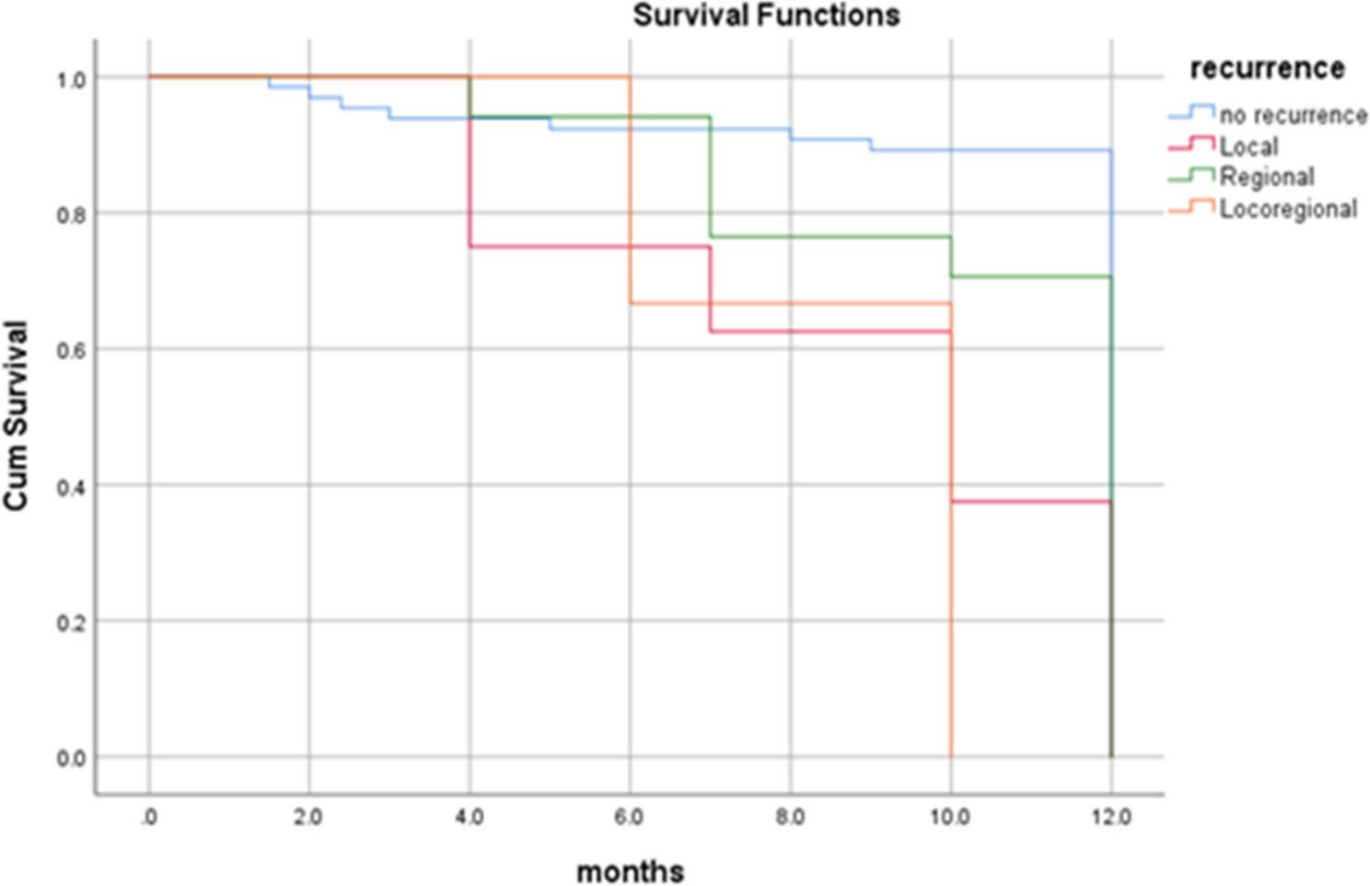
Kaplan Meier survival rate according to types of the recurrences

**Fig. 6:**
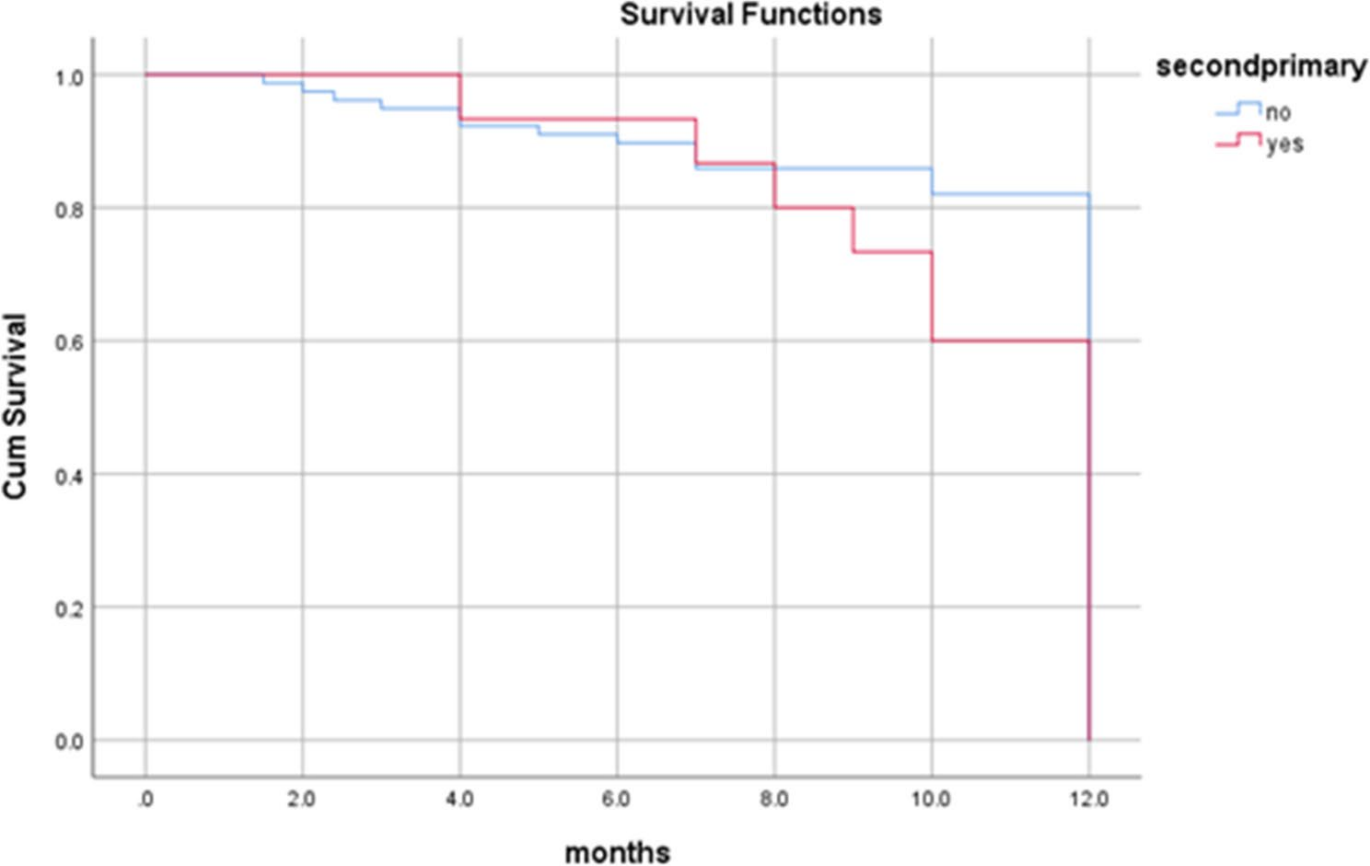
Kaplan Meier survival rate according to presence of the second primary tumors

**Fig. 7:**
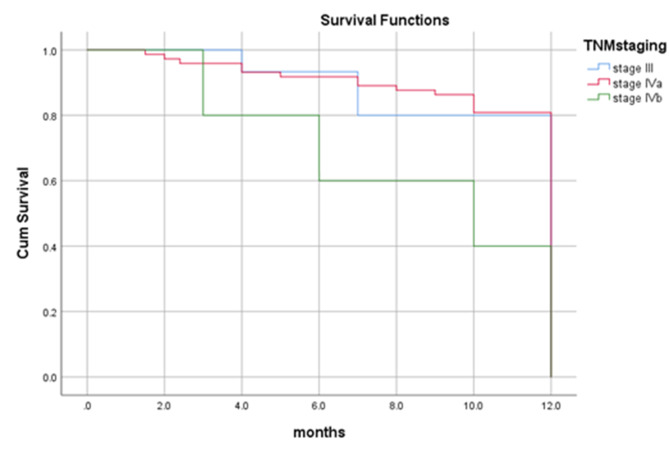
Kaplan Meier survival rate according to the overall tumor stages

**Fig. 8:**
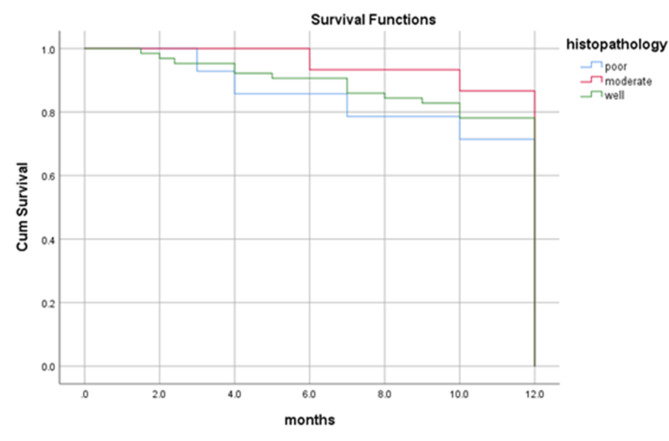
Kaplan Meier survival rate according to the grades of histodifferentiation

**Fig. 9:**
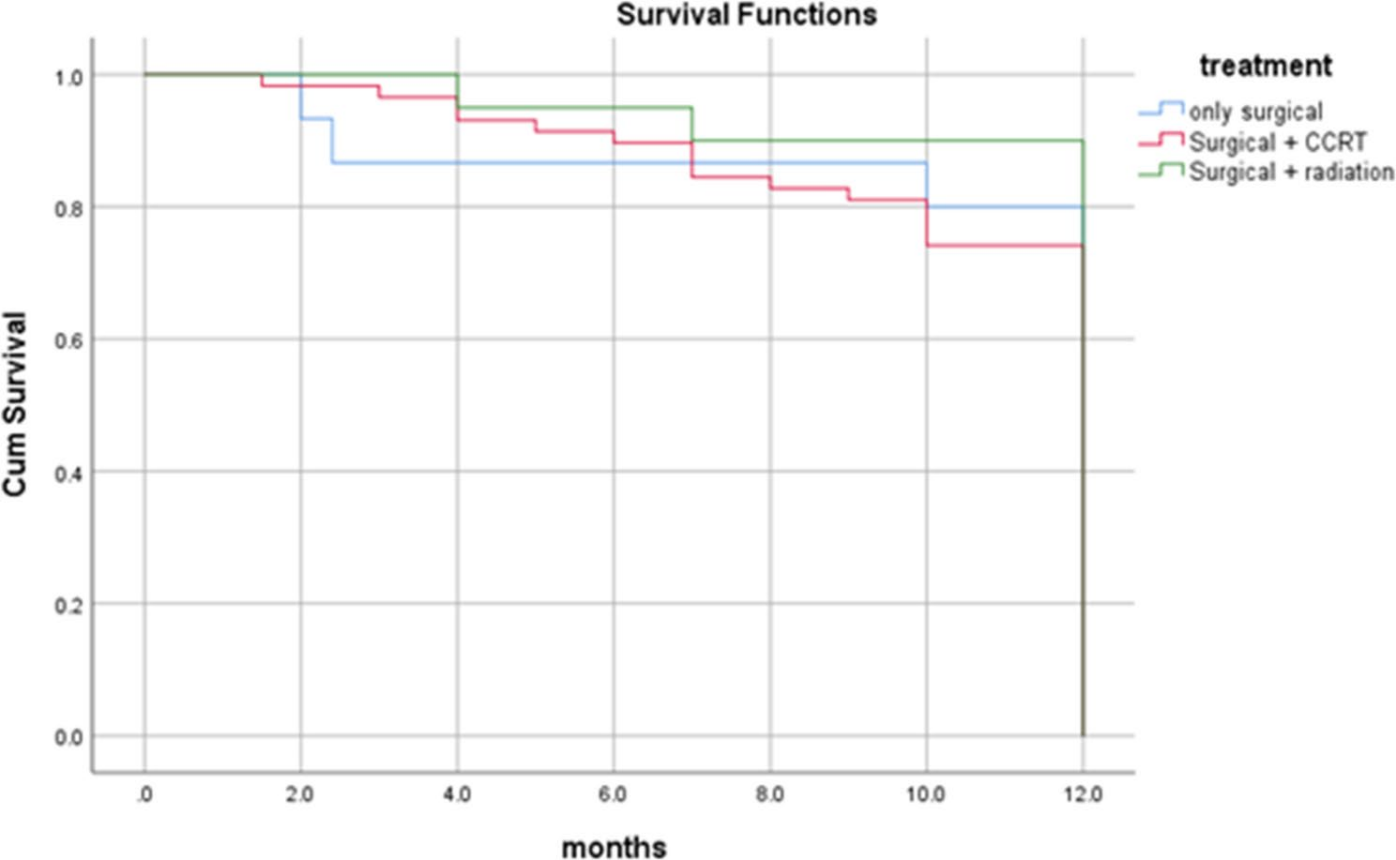
Kaplan Meier survival rates according to the type of treatment modalities

By the end of the study period (3 years and 6 months), more than two-third (75%) of the patients were alive, 66 (71.0%) didn’t suffer from any significant complication. Twenty-three (24.7%) patients died; 10 (10.8%) of them died 8 months following surgery, seven (7.5%) died four months following surgery and six (6.5%) of the patients died one year later. Overall one-year survival rate was 89%, and all the dead patients died by 12 months (Fig. 3). In the present study, the survival rate for overall recurrences was 82%. Dead patients with regional, local and locoregional recurrences died at 12 months with survival rates of 87%, 74% and 72% respectively. Patients with no recurrence have one-year survival rate of 92% (Figs. 4 and 5). Dead patients with second primary tumors died at 11 months with survival rate of 86% unlike those without second primary lesions have lived up for 12 months, with a one-year survival rate of 90% (Fig. 6). In the present study, survival rates for OSCC patients with stages III, IVa and IVb were 90%, 90% and 71% respectively (Fig. 7), and the survival rates for OSCC patients with well, moderate and poor histodifferentiation grades were 89%, 85% and 95% respectively (Fig. 8). According to the treatment modalities, the survival rates for treated OSCC patients by surgery alone, surgery plus CCRT and surgery plus radiotherapy were 87%, 88% and 94% respectively (Fig. 9).

**Table 1:** Bad risky habits

Bad habit	Frequency (%)
Smoking	19 (20.4%)
Alcohol	14 (15.1%)
Toombak dipping	38 (39.9%)

**Table 2:** Oral cavity subsites

Site	Frequency (%)
Gingivobuccal mucosa	69 (74.2%)
Gingivolabial mucosa	58 (62.3%)
Floor of the mouth	26 (28%)
Tongue	23(24.7%)
Maxillary sinus	19 (20.4%)
Retromolar trigone	10 (10.8%)
Palate	8 (8.6%)

**Table 3:** Clinical presentations

	Frequency (%)
Ulcer	93 (100%)
Swelling	18 (19.4%)
Ulcers associated with different mucosal changes	83 (89.2%)
Ulcers associated with by erythematous patches	44 (47.3%)
Pain or numbness	22 (23.7%)

**Table 4:** Overall tumor staging

	Frequency (%)
**Staging**	
Stage IVa [ T4a + N0/N1/N2 M0 ]	69 (74.2%)
Stage III [ T3 + N0/N1 + M0 ]	14 (15%)
Stage IVb [ any T + N3 + M0 ]	4 (4.3%)
Stage IVb [ T4b + any N + M0]	3 (3.2%)
Stage III [ T1/T2 + N1 + M0 ]	3 (3.2%)

**Table 5:** Types of surgeries

	Frequency (%)
Hemiglossectomy	15 (16%)
Partial Glossectomy	7 (7.5%)
1/2 of the Lip	5 (5.4%)
1/3 to 2/3 of the Lip	9 (9.6%)
Hemi-Maxillectomy	10 (10.8%)
Subtotal Maxillectomy	9 (9.7%)
Hemi-mandibulectomy	40 (43.0%)
Subtotal mandibulectomy	13 (14.0%)
Supraomohyoid neck dissection	23(24.7%)
Radical neck dissection	2 (2.2%)
Modified radical neck dissection	91(98%)

**Table 6:** Surgical reconstruction, adjuvant therapy and fate of patients at the end of the study

Adjuvant therapy	
CCRT	58 (62.4%)
Radiation	20 (21.5%)
Surgical reconstruction	13 (14%)
**Type of flap used**	
Nasolabial flap	9 (9.7%)
Deltopectoral flap	4 (4.3%)

**Table 7:** Histopathological differentiation, microscopic depth of invasion, and presence and grading of dysplasia

	Frequency (%)
**Degree of Histopathology Differentiation**	
Well	64 (68.8%)
Moderate	15 (16%)
Poor	14 (15%)
**Microscopic Depth of Invasion [ in mm ]**	
> 4 mm	92 (98.9%)
< 4 mm	1 (1%)
**Presence of histologic grade of dysplasia in field area**	
No	80 (86%)
Yes ( all of them were mild dysplasia)	13 (14%)

**Table 8:** Post-operative histological characteristics

	Frequency (%)
Extra nodal extension	43 (46.2%)
Positive pathologic lymph nodes	58 (62.4%)
Lymphovascular invasion	76 (81.7%)
Perineural invasion	72 (77.4%)
**Status of surgical margins**	
Negative	92 (98.9%)
Positive	1 (1%)

**Table 9:** Characteristics of recurrence and SPTs

	Frequency (%)
Presence of recurrence [ Over All ]	28(30%)
Regional	17 (18.3%)
Local	8 (8.6%)
Loco-regional	3 (3.2%)
Recurrence within the flap	4 (4.3%)
Presence of second primary tumors	14 (15%)

**Table 10:** Logistic regression variables of recurrence

		B	S.E.	Wald	df	Sig.
Step 1^a^	DNA ploidy	0.630	0.576	1.196	1	0.032
	SPF	0.052	0.092	0.324	1	0.042
	TNM staging	0.740	0.801	0.852	1	0.356
	Histodifferentiation	− 0.619	0.464	1.781	1	0.182
	dysplasia	-1.332	1.165	1.307	1	0.253
	Constant	-25.959	40192.992	0.000	1	0.999

**Table 11:** Logistic regression variables of second primary tumors

		B	S.E.	Wald	df	Sig.
Step 1^a^	DNA ploidy	1.386	0.332	17.427	1	0.003
	SPF	0.132	0.080	2.727	1	0.043
	TNM staging	1.589	0.811	3.842	1	0.040
	Histodifferentiation	0.036	0.492	0.005	1	0.941
	dysplasia	− 0.359	0.928	0.149	1	0.699
	Constant	24.758	40193.020	0.000	1	1.000

## Discussion

The hypothesis of genetically altered cells described these cells as to have the ability to spread vigorously beyond the OSCC epithelium, into the clinically and histologically normal tissues, resulting in a wide field change of cancer [4]. On the other hand, the clonally linked malignant cells hypothesis asserts that more than 50% of the clear surgical margins of OSCC cases, and the assessed margins were clear of any tumor cells on the microscopic examination [4].

The present study showed that 99% of the cases in this series were involving multiple regions and only one case was of a single site involvement. In contrast to the previous published studies [3, 11–13], which showed the gingivolabial site as the most frequent site for OSSC in the Sudan, the present study showed the gingivobuccal mucosa was the most frequently involved sites accounting for 74.2% of the patients. This variation may be attributed to the nature of the wide field involvement of multiple regions in almost all the cases of the present study. The involvement of the buccal mucosa in most of the cases might be due to the lateral wide spread of the malignant cells and to its exposure to the carcinogens. In the current study all cases presented lately with advanced stages, it’s extremely crucial to have early recognition of the OSCC cases particularly those with wide field of cancerization and recommending the media to encourage the population to stop the bad habits especially Toombak dipping and Tobacco smoking, additionally educating the dentists about the clinical characteristics of early presentation, diagnosis and treatment of OSCC cases particularly those with clinical characteristics of wide field of cancerization.

In the present study, all the patients had neck dissection as a fundamental surgical procedure, where the vast majority (97.8%) of the patients had modified radical neck dissection followed by supra-omohyoid ND in 24.7% of the cases and only two cases had radical ND. This line of management is ideal and usually adopted by cancer surgeons who are experienced in surgery of OSCC patients [14]. Ninety-two of the cases (98.9%) showed negative margins and only one case had positive margin, indicating a better result than that what was reported by Jain et al. [15]., who showed that 81% of their cases had negative margins, 14.7% had close margins and 4.2% of the cases had positive margins. Getting a good safe margin of an oral lesion is not always an easy task for the surgeon; particularly when the lesion is very extensive and overlapping the structure in the vicinity. For the histopathologist, the surgical margins of OSCC cases in particular, are challenging to be assessed due to many factors including: the multiplex three-dimensional anatomical structures and barriers of the oral cavity regions, the moderate difficulty of holding and labelling of the gross specimen after tissue shrinkage; post-surgery and after fixation, and the microscopic interpretation with its inter and intra-observer subjectivity. In the present study, dysplasia at surgical margins in OSCC cases with clinical wide field of cancerization was identified in only 14% of the cases. The histological dysplasia at the surgical margins was reported in many studies ranging from 2.26 to 46.1% [16]. Although all the cases in the study had clear margins except one case, 30% of the patients developed recurrence, 18.3% of the study sample had regional recurrence in the neck. The local recurrence was followed by locoregional recurrences in 8.6% and 3.2% of the cases respectively. Similar findings were reported by different investigators such as Anderson et al. [17]., and Priya et al. [18]., who found 21% and 22.2% recurrence rates in their OSCC cases, with clear surgical margins respectively.

In the present study, 15% of the cases developed second primary lesions. A similar finding was reported by Cianfriglia F et al., as in their OSCC patients, the incidence rate of SPTs was 14% [19]. Also, Haring VD et al. reported in their study SPTs, a 5-year incidence rate of 13% and a 10-year incidence rate of 21% [20]. These reports, underline the risk of developing SPTs over the years, and emphasizing the importance of a strict and regular follow-up of patients of OSCC, particularly, those with wide field of oral cancerization. Importantly to mention, that all the SPTs in this series have occurred in OSCC patients who were Toombak dippers.

The present study also showed that after one year following surgery, 75% of the overall patients were alive, 71% of them didn’t suffer from any significant complications, while 24.7% of the series died; 10.8% died at 8 months, and 7.5% died at 4 months following surgery. The overall one-year survival rate was 89%. Furthermore, the survival rate for the overall recurrences was 82%. Patients with regional, local and locoregional recurrences had survival rates of 87%, 74% and 72% respectively. Rathan et al., [21] reported a 2-year survival rate of 54% and 41% for cases with stages I and II respectively, and for stage III and stage IV, 18% and 3% survival rates respectively. Different findings were reported by Campion L et al., who showed, an overall survival of 59.9% in one year and 40.7% in two years, and the survival rate of patients who underwent surgical treatment with adjuvant therapy was 84.2% [22]. Wang et al. [23]., in their study showed that the 2- and 5-year survival rates were significantly lower in the recurrence group than in the non-recurrence group (67.6% vs. 88.0%, 31.8% vs. 79.9%, *P* < 0.001). Recently, Sousa et al. [24]., reported that the average survival of their patients with local, regional, and distant recurrence was 12, 5, and 2 months, respectively. Patients with recurrent lesions had lower survival rate (*P* < 0.001) and cases with local recurrent tumors had higher survival rate than those with regional or distant recurrence (*P* = 0.011). All cases with regional and distant recurrence died by the end of the follow-up. Liao et al. [25]., showed that the mean cutoff interval for recurrence was 10 months and late relapse was significantly associated with a higher 5-year disease specific survival and overall survival (*p* < 0.0001). Recently, Eltohami and Suleiman [26] investigated that local and locoregional recurrences as significant prognosticators of OSCC associated with wide field of cancerization in cases associated with variable changes in the oral mucosa, specifically Toombak users.

Elsheikh M [27]. has shown that the overall 1, 2, 3 and 5-year overall survival rates were 51%, 39.9%, 36.7% and 34% respectively, and the histological degree of differentiation, tumor size and nodal metastasis were identified as the most significant independent prognosticators. The one-year survival rate in the present study is very high mounting to 89% compared to the 51% was reported by Elsheikh M. The study material of Elsheikh were collected from all the records of the Surgical Units in Khartoum Teaching Dental Hospital (KTDH), while the material of the present study was collected from the records of one Surgical Unit known for oral cancer surgery.

The present study, showed that the patients with SPTs had a survival rate of 86%, while the survival rate in patients with no SPTs was 90% Yung-An et al. [28], found that in their HNSCC cases, patients who developed metachronous head and neck SPTs in early stages and who underwent curative treatment had a better survival rate than patients who developed synchronous SPTs in non-head and neck regions with an advanced disease and underwent palliative treatment. They concluded that, early stage SCC patients who developed metachronous SPTs showed a better survival rate when the SPTs occurred in the head and neck region and treated surgically. Laurent H. and colleagues reported that, the one-year overall survival after the diagnosis of the SPTs was 72% in their patients. These authors found that the anatomical localization of the SPTs significantly affected the survival after the development of the second tumors [29]. However, Xavier L et al. [30], stated that the survival outcome was more promising in SPTs of the head and neck region, reporting a one-year survival of 84%. On other-hand hand, SPTs of the lung or esophagus showed a significantly worse outcome, with a one-year survival of only 50% and 29%, respectively, and the SPTs in sites other than the aerodigestive tract demonstrated a one-year survival of 68%.

## Conclusion

This study highlights the high prevalence of advanced-stage oral cancer patients, particularly among Toombak dippers with no cases present with early tumor stage. It underscores that even with clear surgical margins and adjuvant concurrent chemoradiation therapy, recurrence and second primary tumours can still occur. Nevertheless, the overall one-year survival rate was high at 89%. These findings emphasize the importance of close and regular follow-up, especially for patients with a wide field of oral cancerization, and the need to consider multiple factors in assessing survival outcomes.

## Data Availability

The datasets used and/or analysed during the current study are available from the corresponding author upon reasonable request.
